# Risk stratification in patients with structurally normal hearts: Does fibrosis type matter?

**DOI:** 10.1371/journal.pone.0295519

**Published:** 2023-12-20

**Authors:** Katarzyna E. Gil, Katarzyna Mikrut, Jan Mazur, Ann Lowery Black, Vien T. Truong, Suzanne Smart, Karolina M. Zareba

**Affiliations:** 1 The Ohio State University Division of Cardiovascular Medicine, Columbus, OH, United States of America; 2 Advocate Heart Institute, Advocate Lutheran General Hospital, Chicago, IL, United States of America; 3 University of Cincinnati College of Medicine, Cincinnati, OH, United States of America; 4 The Ohio State University College of Medicine, Columbus, OH, United States of America; 5 Department of Internal Medicine, Nazareth Hospital, Philadelphia, PA, United States of America; 6 Dorothy M. Davis Heart and Lung Research Institute, The Ohio State University, Columbus, OH, United States of America; Baruch Padeh Medical Center Poriya, ISRAEL

## Abstract

**Objectives:**

The study sought to assess the prognostic significance of nonischemic myocardial fibrosis (MF) on cardiovascular magnetic resonance (CMR)–both macroscopic MF assessed by late gadolinium enhancement (LGE) and diffuse microscopic MF quantified by extracellular volume fraction (ECV)–in patients with structurally normal hearts.

**Background:**

The clinical relevance of tissue abnormalities identified by CMR in patients with structurally normal hearts remains unclear.

**Methods:**

Consecutive patients undergoing CMR were screened for inclusion to identify those with LGE imaging and structurally normal hearts. ECV was calculated in patients with available T1 mapping. The associations between myocardial fibrosis and the outcomes of all-cause mortality, new-onset heart failure [HF], and an arrhythmic outcome were evaluated.

**Results:**

In total 525 patients (mean age 43.1±14.2 years; 30.5% males) were included. Over a median follow-up of 5.8 years, 13 (2.5%) patients died and 18 (3.4%) developed new-onset HF. Nonischemic midwall /subepicardial LGE was present in 278 (52.9%) patients; isolated RV insertion fibrosis was present in 80 (15.2%) patients. In 276 patients with available T1 mapping, the mean ECV was 25.5 ± 4.4%. There was no significant association between LGE and all-cause mortality (HR: 1.36, CI: 0.42–4.42, p = 0.61), or new-onset HF (HR: 0.64, CI: 0.25–1.61, p = 0.34). ECV (per 1% increase) correlated with all-cause mortality (HR: 1.19, CI: 1.04–1.36, p = 0.009), but not with new-onset HF (HR: 0.97, CI: 0.86–1.10, p = 0.66). There was no significant association between arrhythmic outcomes and LGE (p = 0.60) or ECV (p = 0.49). In a multivariable model after adjusting for covariates, ECV remained significantly associated with all-cause mortality (HR per 1% increase in ECV: 1.26, CI: 1.06–1.50, p = 0.009).

**Conclusion:**

Nonischemic LGE in patients with structurally normal hearts is common and does not appear to be associated with adverse outcomes, whereas elevated ECV is associated with all-cause mortality and may be an important risk stratification tool.

## Introduction

Developments in myocardial tissue characterization by cardiovascular magnetic resonance imaging (CMR) have contributed to a rapid increase in its utilization in a variety of clinical indications [1]. The resultant identification of tissue abnormalities, including myocardial fibrosis (MF), in patients with otherwise structurally normal hearts, has raised questions about their clinical significance. MF can occur at the macroscopic (replacement or focal fibrosis) or microscopic (reactive or diffuse interstitial fibrosis) level [2].

There is limited data evaluating the prognostic significance of nonischemic macroscopic MF as assessed by late gadolinium enhancement (LGE) imaging in patients with preserved left ventricular ejection fraction (LVEF) and no structural abnormalities (such as left ventricular [LV] hypertrophy or dilation) [3, 4]. Subendocardial ischemic pattern LGE and its association with all-cause mortality is the most widely studied LGE pattern in patients with no known cardiovascular disease [4]. Nonischemic LGE is an established adverse prognostic indicator in patients with various cardiomyopathies [1, 5–10]. However, uncertainty regarding the significance of nonischemic LGE in those with structurally normal hearts may lead to ambiguity in clinical management, anxiety of patients and physicians, or result in redundant clinical testing and increased healthcare costs.

Data on the prognostic significance of interstitial MF quantified by extracellular volume fraction (ECV) in patients with structurally normal hearts is scarce. ECV elevation is nonspecific and results predominantly from excess accumulation of collagen in the interstitium in the absence of confounding conditions such as myocardial edema or amyloidosis [11–13]. Since diffuse microscopic interstitial MF results in adverse myocardial remodeling, its identification in patients with preserved LVEF and no structural heart disease may enable diagnosis at a preclinical and potentially reversible stage [2, 12, 14]. Higher ECV is associated with death and hospitalization for heart failure (HF) in heterogeneous cohorts of all-comers referred for CMR [11, 15–18]. The association is even stronger for patients with type 2 diabetes and persists when LGE segments are excluded [11, 16, 19]. Importantly, ECV has been shown to be more strongly associated with adverse outcomes than nonischemic LGE, and at least as strong as LVEF [15, 18]. These studies include patients with both ischemic and nonischemic LGE across a spectrum of LV function [15, 18]. Similar associations were found in patients with coronary artery disease, HF with preserved EF, and dilated cardiomyopathy [17–21].

The overall goal of our study was to evaluate the prognostic utility of nonischemic MF in patients with structurally normal hearts. Specifically, we sought to evaluate both nonischemic LGE fibrosis and interstitial fibrosis by ECV and their associations with outcomes in patients with structurally normal hearts. Based on prior studies, we hypothesized that the presence nonischemic LGE in this cohort would not be associated with adverse outcomes; however, the presence of interstitial fibrosis by ECV, an objective and reproducible metric of interstitial expansion and myocardial architecture, may help stratify patients into higher and lower risk cohorts.

## Materials and methods

### Patients

This is a retrospective study assessing the prognostic significance of MF in patients with preserved LVEF and no structural cardiac abnormalities. The study was approved by Ohio State University’s Institutional Review Board who waived informed consent. The study was conducted between 2017 and 2023. Authors had access to identifying information during the collection of data, which was then removed prior to data analysis. Consecutive patients undergoing clinical CMR on 1.5T scanners (Magnetom Avanto and Espree, Siemens Medical Solutions, Erlangen, Germany) between May 2010 and May 2017 were evaluated for inclusion. The data time period of 2010 to 2017 was chosen to allow us to query all available consecutive CMR exams at our institution, and at the same time provide long term follow up of at least 5 years. Inclusion criteria were LVEF ≥50%, age ≥18 years old, and the presence of LGE imaging. Although contrast administration with LGE imaging was a requirement for inclusion (given the premise to evaluate the association of myocardial fibrosis and outcomes), T1 mapping including ECV calculation was not a requirement for inclusion as it was not routinely performed on all patients during the study period, particularly prior to 2014. The electronic medical record was reviewed for demographic and clinical data, and cardiac testing (electrocardiography, Holter monitoring, stress testing, echocardiography, coronary computed tomography angiography, single photon emission computed tomography, and left heart catheterization).

**Fig 1:**
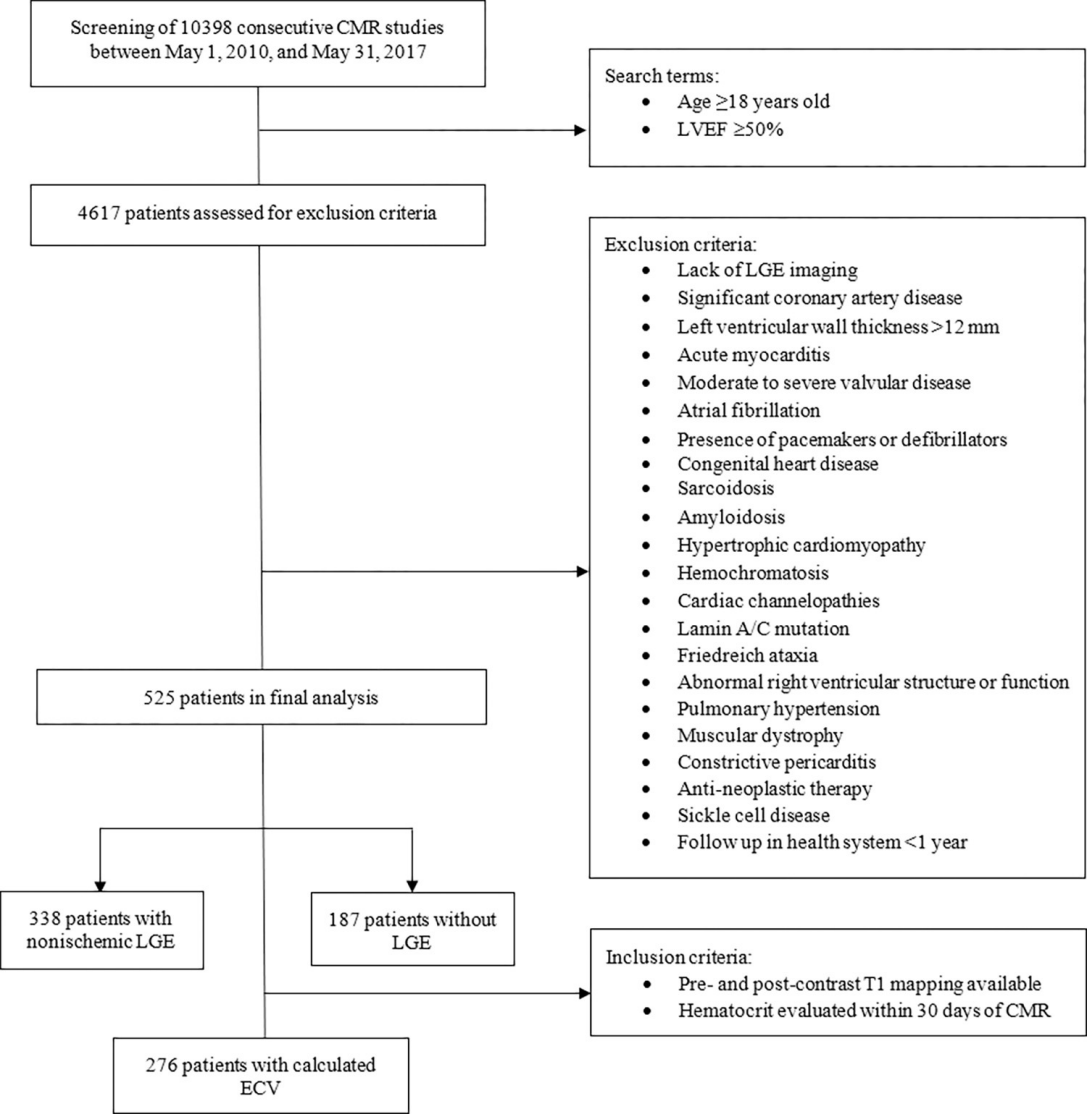
Patient flow diagram. Abbreviations: CMR, cardiovascular magnetic resonance imaging; ECV, extracellular volume fraction; LVEF, left ventricular ejection fraction; LGE, late gadolinium enhancement imaging.

Exclusion criteria were aimed at creating a low-risk cohort without structural heart disease and without findings or disease processes known to be associated with worse clinical outcomes. The exclusion criteria were as follows: significant coronary artery disease (infarct LGE pattern, positive stress testing, obstructive lesions on left heart catheterization >70%, or prior coronary intervention); LV hypertrophy >12 mm; acute myocarditis; moderate or severe valvular disease; atrial fibrillation; congenital heart disease; sarcoidosis, amyloidosis; hypertrophic cardiomyopathy, hemochromatosis; cardiac channelopathies; lamin A/C mutation; abnormal right ventricular structure/function; pulmonary hypertension; Friedreich ataxia; dystrophies; constrictive pericarditis; anti-neoplastic therapy; and sickle cell disease. Additionally, patients with pacemakers and defibrillators were excluded (Fig 1). To ensure comprehensive outcomes data, only patients who were followed within The Ohio State University health system for at least one year after the CMR exam were included.

### CMR image acquisition & analysis

**Fig 2:**
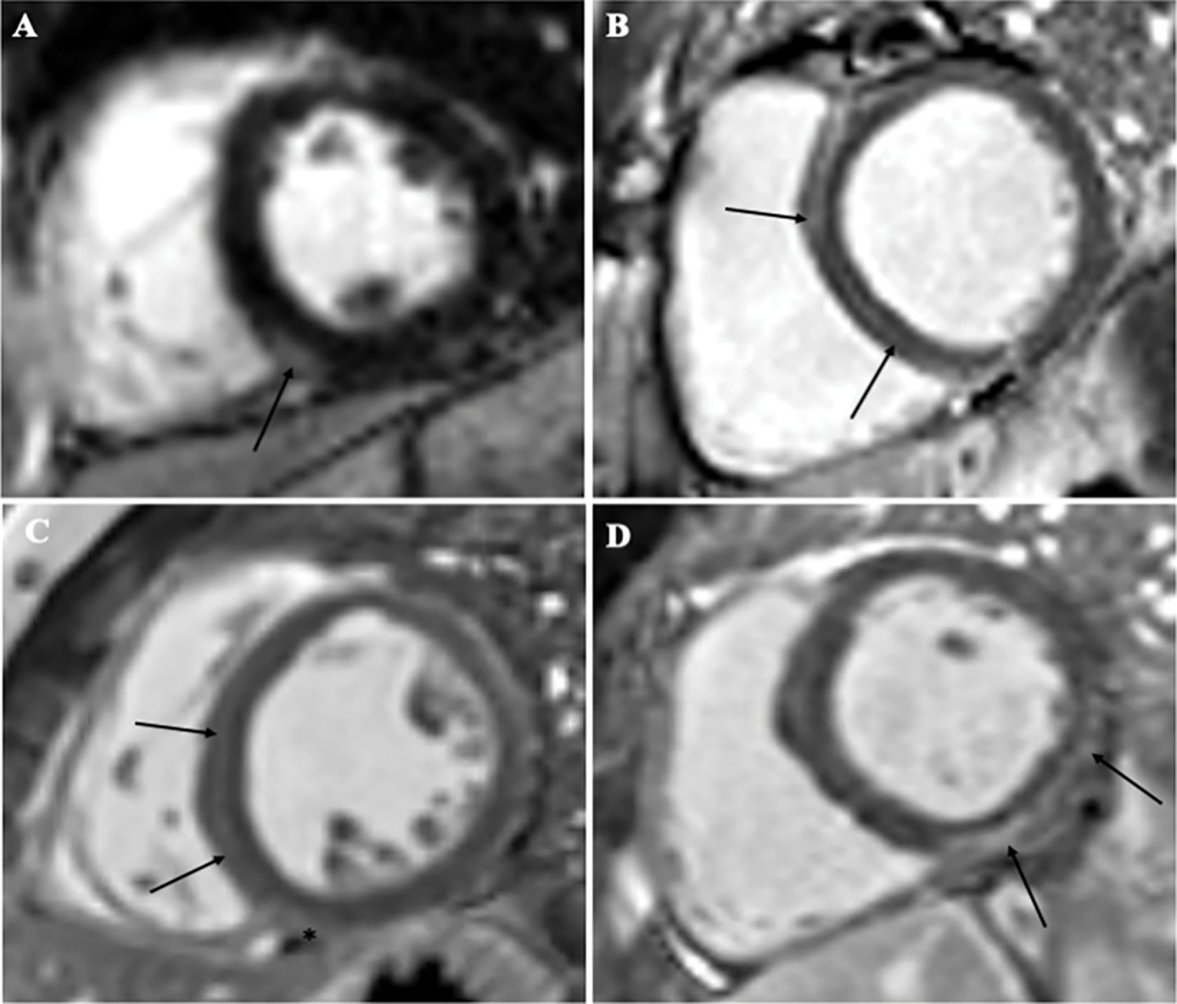
Fibrosis in patients with structurally normal hearts. (A) Fibrosis in the inferior right ventricular insertion point (RVIP) (arrow). (B) Midmyocardial fibrosis in the septum (arrows). (C) Midmyocardial fibrosis in the septum (arrows) with RVIP fibrosis (asterisk). (D) Subepicardial fibrosis in the basal inferior/inferolateral walls (arrows).

CMR images were acquired using standardized protocols including cine imaging, pre-contrast and post-contrast T1 mapping, and LGE imaging [22]. LV volumes and LVEF were measured from short-axis stacks of cine frames that covered the LV. The left atrial volume index (LAVI) was calculated using the biplane area-length method [23]. Native and post-contrast myocardial T1 values (Modified Look-Locker Inversion Recovery) were measured within the septum on the mid short axis (SAX) maps and ECV was calculated using the standard formula [13]. The septal region of interest was chosen based on prior data supporting its use and included regions of LGE if present. Hematocrit (within 30 days) was utilized for ECV calculation. The presence, pattern, and extent of LGE was assessed by two level 3 CMR readers blinded to outcomes data. LGE patterns were as follows: isolated right ventricular insertion point [RVIP], midwall, and subepicardial (Fig 2). Extent of LGE was reported using the seventeen segment American Heart Association [AHA] classification [24]. S1 Table provides CMR sequence details.

### Follow-Up & outcomes

Patient follow-up was performed by review of the electronic medical record and vital status. Follow-up duration was calculated from the date of CMR. The primary outcomes were all-cause mortality and new-onset HF. The electronic medical record was reviewed in detail to ascertain cause of death. Cardiovascular death was defined as sudden cardiac death, heart failure, stroke, or thromboembolism. The lack of precise cause of death was not an exclusion criterion. New-onset HF was defined as new diagnosis of HF documented by a physician requiring diuretic therapy. A secondary arrhythmic outcome was defined as ventricular tachycardia (VT), ventricular fibrillation, VT ablation, or appropriate implantable cardioverter defibrillator (ICD) intervention.

### Statistical analysis

Categorical data are expressed as frequency with percentage, and comparison between groups was performed using the chi-square test or Fisher exact test. The distribution of continuous variables was assessed using skewness, kurtosis, visual inspection of the histogram, and QQ plot. Continuous variables are displayed as mean ± standard deviation (SD) for normal distribution or presented as median (interquartile range) for non-normal distribution. T test or Mann–Whitney U test was performed to compare differences between two groups for normally and non-normally distributed variables, respectively. Kaplan-Meier survival curves were drawn to assess differences between groups for time to event data. Time zero was defined as the date of CMR study. A Cox regression model was used to assess the relationship of MF with clinical outcomes. Clinical data (age, gender, LGE) were included in the multivariable model. To test the robustness of association of ECV and all-cause mortality, the additional sensitivity analyses were planned: the first was using the bootstrap method with 100 resampling technique to estimate 95% percentile bootstrap confidence intervals, the second was assessing the association of ECV and all-cause mortality in the different multivariable models. Furthermore, the optimal cutoff value for ECV and outcomes of mortality was determined using maximally selected rank statistics, which take time-to-event into account [25]. Proportionality assumptions of the Cox regression model were assessed by Schoenfeld residuals. The deviance residuals and the dfbeta values were used to examine influential observations. Martingale residuals against continuous predictors were applied to assess nonlinearity and the functional form of predictors. Hazard ratios are presented as mean and 95% confidence intervals. Two-sided p values <0.05 were considered statistically significant. Statistical analyses were performed using IBM SPSS Statistics for Windows, version 22.0 (IBM Corp., Armonk, N.Y., USA) and R software, version 4.0.3 (The R Foundation, Vienna, Austria) with the “readxl”, “tidyverse”, “table1”, “compareGroups”, “naniar”, “survival”, and “survminer” packages.

## Results

### Baseline characteristics

Of 10,398 patients undergoing CMR between May 2010 and May 2017, a total of 525 patients were included in the analysis (Fig 1). The mean age of the cohort was 43.1 ± 14.2 years, with 30.5% being male (Table 1). Scan indications included: arrhythmia/palpitations/syncope (45.1%), chest pain/dyspnea (29.3%), myocarditis/pericarditis (10.9%), aortic valve/aortic assessment (7.0%), and family history of cardiomyopathy or sudden cardiac death (6.3%). Nonischemic midwall / subepicardial LGE was present in 278 (52.9%) patients, while isolated RV insertion point fibrosis was present in 80 (15.2%) patients–in total 338 patients exhibited any nonischemic LGE. Data to calculate ECV was available in 276 (52.6%) patients (Table 2) with a mean ECV of 25.5 ± 4.4%.

**Table 1:** Baseline clinical and imaging characteristics based on presence of nonischemic late gadolinium enhancement.

Characteristic	Whole cohort (n = 525)	LGE negative (n = 187)	LGE positive (n = 338)	P value
Age, mean (SD), y	43.1 ± 14.2	42.0 ± 14.1	43.8 ± 14.2	0.18
Male, No. (%)	159 (30.5)	29 (15.5)	130 (38.9)	< 0.001
BSA, mean (SD), m^2^	1.93 ± 0.27	1.86 ± 0.27	1.97 ± 0.26	< 0.001
BMI, mean (SD), kg/m^2^	28.7 ± 7.7	28.2 ± 8.4	29.0 ± 7.2	0.25
Hypertension, No. (%)	177 (33.7)	55 (29.4)	122 (36.1)	0.12
Hyperlipidemia, No. (%)	148 (28.2)	39 (20.9)	109 (32.2)	0.005
Diabetes mellitus, No. (%)	44 (8.4)	16 (8.6)	28 (8.3)	0.91
Nonobstructive coronary artery disease, No. (%)	38 (7.2)	13 (7.0)	25 (7.4)	0.85
Stroke, No. (%)	10 (1.9)	4 (2.1)	6 (1.8)	0.75
Heart failure history, No. (%)	37 (7.0)	12 (6.4)	25 (7.4)	0.68
***Pharmacotherapy*, *No*. *(%)***				
Antiplatelets	138 (26.3)	38 (20.3)	100 (29.6)	0.02
Beta blockers	176 (33.5)	55 (29.4)	121 (35.8)	0.14
ACE-inhibitors	74 (14.1)	19 (10.2)	55 (16.3)	0.054
Angiotensin receptor blockers	38 (7.2)	11 (5.9)	27 (8.0)	0.37
Antiarrhythmics	42 (8.0)	16 (8.6)	26 (7.7)	0.73
Diuretics	69 (13.1)	17 (9.1)	52 (15.4)	0.04
Mineralocorticoid Receptor Antagonists	11 (2.1)	4 (2.1)	7 (2.1)	0.96
***Scan Indications*, *No*. *(%)***				
Arrhythmia/palpitations/syncope	237 (45.1)	81 (43.3)	156 (46.2)	0.53
Chest pain/Dyspnea	154 (29.3)	66 (35.3)	88 (26.0)	0.03
Myocarditis/pericarditis	57 (10.9)	23 (12.3)	34 (10.1)	0.44
Aortic valve/aortic assessment	37 (7.0)	10 (5.3)	27 (8.0)	0.26
Family history of cardiomyopathy/SCD	33 (6.3)	10 (5.3)	23 (6.8)	0.51
Other	74 (14.1)	24 (12.8)	50 (14.8)	0.54
***CMR parameters*, *mean (SD)***				
LVEDVI, ml/m^2^	69.7 ± 14.9	68.5 ± 14.2	70.3 ± 15.2	0.18
LVESVI, ml/m^2^	26.0 ± 7.8	25.5 ± 7.2	26.3 ± 8.1	0.25
LVEF, %	63.0 ± 6.4	63.0 ± 6.0	63.0 ± 6.6	0.98
LVSVI, ml/m^2^	43.6 ± 9.4	43.0 ± 9.1	44.0 ± 9.6	0.25
LAVI, ml/m^2^	39.5 ± 10.6	38.9 ± 10.9	39.8 ± 10.4	0.36
LGE presence, No. (%)	338 (64.3)	0	338 (100)	
Isolated RVIP, No. (%)			80 (23.7)	
LGE midwall, No. (%)			253 (74.6)	
LGE subepicardial, No. (%)			25 (7.4)	
AHA segments with LGE, median (IQR)			2 (1–2)	
Most frequently involved AHA segments, No. (%)				
Basal inferoseptum			214 (63.3)	
Mid inferoseptum			201 (59.5)	
Basal inferolateral wall			76 (22.5)	

Abbreviations: ACE, angiotensin-converting enzyme; AHA, American Heart Association; BMI, body mass index; BSA, body surface area; CMR, cardiovascular magnetic resonance imaging; IQR, interquartile range; LAVI, left atrial volume index; LGE, late gadolinium enhancement; LVEDVI, left ventricular end-diastolic volume index; LVEF, left ventricular ejection fraction; LVESVI, left ventricular end-systolic volume index; LVSVI, left ventricular stroke volume index; RVIP, right ventricular insertion point; SCD, sudden cardiac death; SD, standard deviation.

**Table 2:** Comparison of cohorts without and with calculated extracellular volume fraction.

Characteristic	Sub-cohort without calculated ECV (n = 249)	Sub-cohort with calculated ECV (n = 276)	P value
Age, mean (SD), y	44.6 ± 14.3	41.8 ± 14.0	0.22
Male, No. (%)	78 (31.3)	81 (29.7)	0.70
BSA, mean (SD), m^2^	1.95 ± 0.28	1.92 ± 0.26	0.21
BMI, mean (SD), kg/m^2^	29.1 ± 8.4	28.3 ± 6.9	0.20
Hypertension, No. (%)	91 (36.5)	86 (31.2)	0.22
Hyperlipidemia, No. (%)	86 (34.4)	62 (22.5)	0.003
Diabetes mellitus, No. (%)	22 (8.8)	22 (8.0)	0.74
Nonobstructive coronary artery disease, No. (%)	20 (8.0)	18 (6.5)	0.52
Stroke, No. (%)	5 (2.0)	5 (1.8)	0.88
Heart failure history, No. (%)	14 (5.6)	23 (8.3)	0.22
***Pharmacotherapy*, *No*. *(%)***			
Antiplatelets	71 (28.5)	67 (24.3)	0.29
Beta blockers	76 (30.5)	100 (36.2)	0.15
ACE-inhibitors	45 (18.0)	29 (10.5)	0.01
Angiotensin receptor blockers	19 (7.6)	19 (6.9)	0.76
Antiarrhythmics	18 (7.2)	24 (8.7)	0.52
Diuretics	40 (16.0)	29 (10.5)	0.07
Mineralocorticoid Receptor Antagonists	8 (3.2)	3 (1.1)	0.13
***Scan Indications*, *No*. *(%)***			
Arrhythmia/palpitations/syncope	75 (30.1)	162 (58.7)	< 0.001
Chest pain/Dyspnea	100 (40.2)	54 (19.6)	< 0.001
Myocarditis/pericarditis	21 (8.4)	36 (13.1)	0.08
Aortic valve/aortic assessment	35 (14.0)	2 (0.7)	< 0.001
Family history of cardiomyopathy/SCD	11 (4.4)	22 (8.0)	0.09
Other	36 (14.4)	39 (14.1)	0.85
***CMR parameters*, *mean (SD)***			
LVEDVI, ml/m^2^	67.9 ± 16.4	71.1 ± 13.2	0.02
LVESVI, ml/m^2^	24.7 ± 8.0	27.2 ± 7.4	< 0.001
LVEF, %	64.1 ± 6.4	62.0 ± 6.2	< 0.001
LVSVI, ml/m^2^	43.3 ± 10.6	43.9 ± 8.2	0.44
LAVI, ml/m^2^	38.9 ± 11.4	39.9 ± 9.9	0.30
LGE presence, No. (%)	167 (67.1)	171 (62.2)	0.27
Isolated RVIP, No. (%)	45 (18.1)	35 (12.7)	0.09
LGE midwall, No. (%)	118 (47.4)	134 (48.7)	0.73
LGE subepicardial, No. (%)	12 (4.8)	13 (4.8)	0.97
Hematocrit (%)	39.8 ± 4.7	39.7 ± 4.9	0.97
Pre-contrast myocardial T1, mean (SD), ms		990.8 ± 46.9	
Post-contrast myocardial T1, mean (SD), ms		423.1 ± 49.7	
ECV, mean (SD), %		25.5 ± 4.4	

Abbreviations: ACE, angiotensin-converting enzyme; BMI, body mass index; BSA, body surface area; CMR, cardiovascular magnetic resonance imaging; ECV, extracellular volume fraction; LAVI, left atrial volume index; LGE, late gadolinium enhancement; LVEDVI, left ventricular end-diastolic volume index; LVEF, left ventricular ejection fraction; LVESVI, left ventricular end-systolic volume index; LVSVI, left ventricular stroke volume index; RVIP, right ventricular insertion point; SCD, sudden cardiac death; SD, standard deviation.

Patients with nonischemic LGE were more likely to be male, have hyperlipidemia, and be on antiplatelet agents and diuretics as compared to those without LGE. There were no other significant differences between groups in age, comorbidities, and pharmacotherapy. There were no significant differences in LV volumes and function or left atrial volume in patients with vs without LGE.

As not all patient in the cohort had a calculated ECV (due to T1 mapping not being a standard inclusion in our imaging protocols prior to the year 2014), the two sub-cohorts (those with and without a calculated ECV) were compared (Table 2). The main differences between the two cohorts included the presence of hyperlipidemia, scan indications, as well as slightly larger LV volumes and lower LV function in the cohort with a calculated ECV. The higher prevalence of hyperlipidemia in the cohort without a calculated ECV may relate to different scan indications (patients without a calculated ECV were more often referred for the evaluation of chest pain and dyspnea rather than arrhythmias, palpitations, and syncope).

#### Myocardial fibrosis characteristics

Of the patients who exhibited LGE, the most common pattern was midwall fibrosis (74.6%), followed by isolated RVIP fibrosis (23.7%), and subepicardial (7.4%) LGE pattern (Table 1). The median number of involved AHA segments was 2, with the most commonly involved segments being the basal (63.3%) and mid inferoseptum (59.5%) extending beyond RVIP, as well as the basal inferolateral wall (22.5%). When comparing the two sub-cohort with vs without a calculated ECV, there were no significant differences in LGE presence, location, and extent (Table 2).

#### Association of fibrosis and outcomes

**Fig 3:**
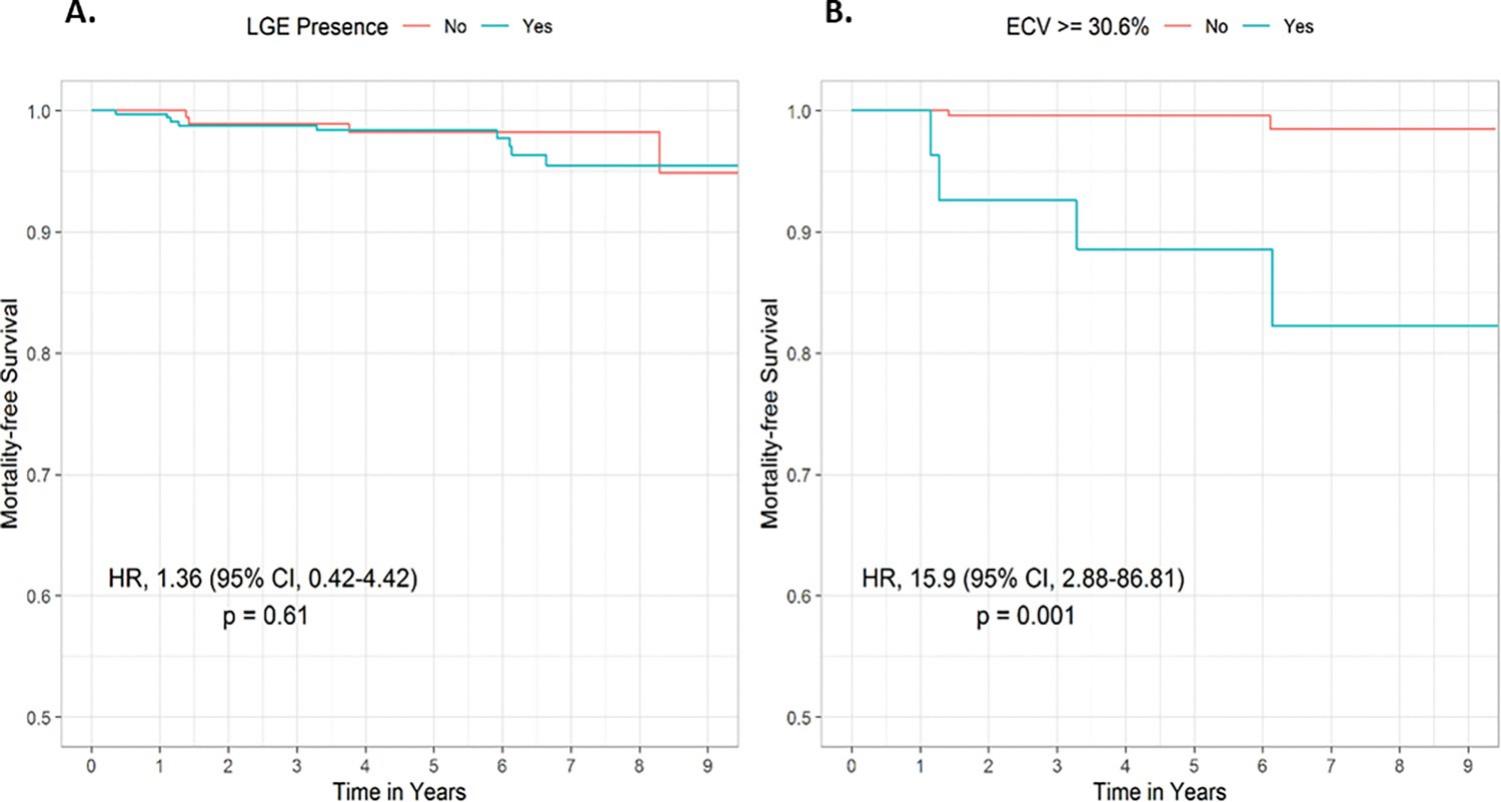
Association between myocardial fibrosis and outcomes. There was no association between LGE and all-cause mortality (Panel A). There was an association between ECV and all-cause mortality (Panel B). Abbreviations: CI, Confidence Interval; ECV, extracellular volume fraction; HR, Hazard Ratio; LGE–late gadolinium enhancement.

Over a median follow-up of 5.8 years (IQR: 3.9–7.6), 13 (2.5%) patients died and 18 (3.4%) developed new-onset HF. Among the sub-cohort with calculated ECV, 6 (2.2%) patients died and 13 (4.7%) developed new-onset HF. Based on review of the electronic medical record, cardiovascular death was attributed as the cause in 5 of 13 patients, while the remaining 8 patients had an unknown cause of death. There were no significant differences in all-cause mortality (HR: 1.36, 95% CI: 0.42–4.42, p = 0.61), or new-onset HF (HR: 0.64, 95% CI: 0.25–1.61, p = 0.34) in patients with vs without LGE (Table 3; Fig 3, Panel A). Within the LGE positive group, there was no association between the primary outcomes and the number or location of involved AHA segments (S2 and S3 Tables).

**Table 3:** Associations between myocardial fibrosis and clinical outcomes.

	HR	95% CI	P value
**LGE presence**			
All-cause Mortality	1.36	0.42–4.42	0.61
New-Onset HF	0.64	0.25–1.61	0.34
Arrhythmic Outcome	1.24	0.56–2.76	0.60
**ECV (per 1% increase)**			
All-cause Mortality	1.19	1.04–1.36	0.009
New-Onset HF	0.97	0.86–1.10	0.66
Arrhythmic Outcome	0.97	0.88–1.06	0.49

Abbreviations: CI, Confidence Interval; ECV, Extracellular Volume; HR, Hazard Ratio; HF, Heart Failure; LGE, Late Gadolinium Enhancement.

An association was found between ECV and all-cause mortality (HR per 1% increase in ECV: 1.19, CI: 1.04–1.36, p = 0.009) (Table 3). There was no significant association between ECV and new-onset HF (HR: 0.97, CI: 0.86–1.10, p = 0.66). Limited events in this young low risk population allowed adjusting for only several covariates. ECV remained significantly associated with all-cause mortality after adjusting for age, gender, and LGE presence (HR per 1% increase in ECV: 1.26, CI: 1.06–1.50, p = 0.009). (Table 4). Sensitivity analysis using the bootstrap method confirmed that there was a significant association between ECV and all-cause mortality (95% percentile bootstrap CI, 1.01–2.79, p = 0.04). Furthermore, the association of ECV and all-cause mortality remained significant in different multivariable models (S4 Table). The optimal ECV cutoff for all-cause mortality using maximally selected rank statistics was 30.6% (S1 Fig). Our study demonstrated that having an ECV≥30.6% was significantly associated with increased mortality compared to those with an ECV<30.6% (HR, 15.9, 95% CI, 2.88–86.81, p = 0.001) (Fig 3, Panel B). The proportional hazards assumption of the Cox model is met by statistical tests and graphical diagnostics using scaled Schoenfeld residuals (S2 Fig).

**Table 4:** Multivariable analysis of the association between ECV and all-cause mortality.

Model	HR	95% CI	P value
ECV (per 1% increase)	1.26	1.06–1.50	0.009
Age (per 1 year)	1.04	0.97–1.11	0.28
Male	0.66	0.07–5.83	0.71
LGE presence	5.58	0.60–52.15	0.13

Abbreviations: CI, Confidence Interval; ECV, Extracellular Volume; LGE, HR, Hazard Ratio; Late Gadolinium Enhancement

The arrhythmic outcome was met by 27 (5.1%) patients, including 25 (9.1%) patients in the sub-cohort with calculated ECV. A total of 16 patients developed VT or ventricular fibrillation. VT ablation was performed in 15 patients. One patient had an appropriate ICD intervention. There was no association between arrhythmic outcomes and LGE presence or ECV.

To further explore the relationship between nonischemic LGE and outcomes, we performed a subanalysis of patients with midwall and subepicardial LGE (excluding 80 patients with isolated RVIP fibrosis). We compared patients with midwall (n = 253) and subepicardial (n = 25) LGE with patients without LGE (n = 187). There were no significant differences in all-cause mortality (HR: 1.78, 95% CI: 0.58–5.44, p = 0.31), new-onset HF (HR: 0.59, 95% CI: 0.22–1.57, p = 0.29), or the arrhythmic outcome (HR: 1.05, 95% CI: 0.50–2.24, p = 0.89) in patients with vs without midwall/subepicardial LGE.

## Discussion

**Fig 4:**
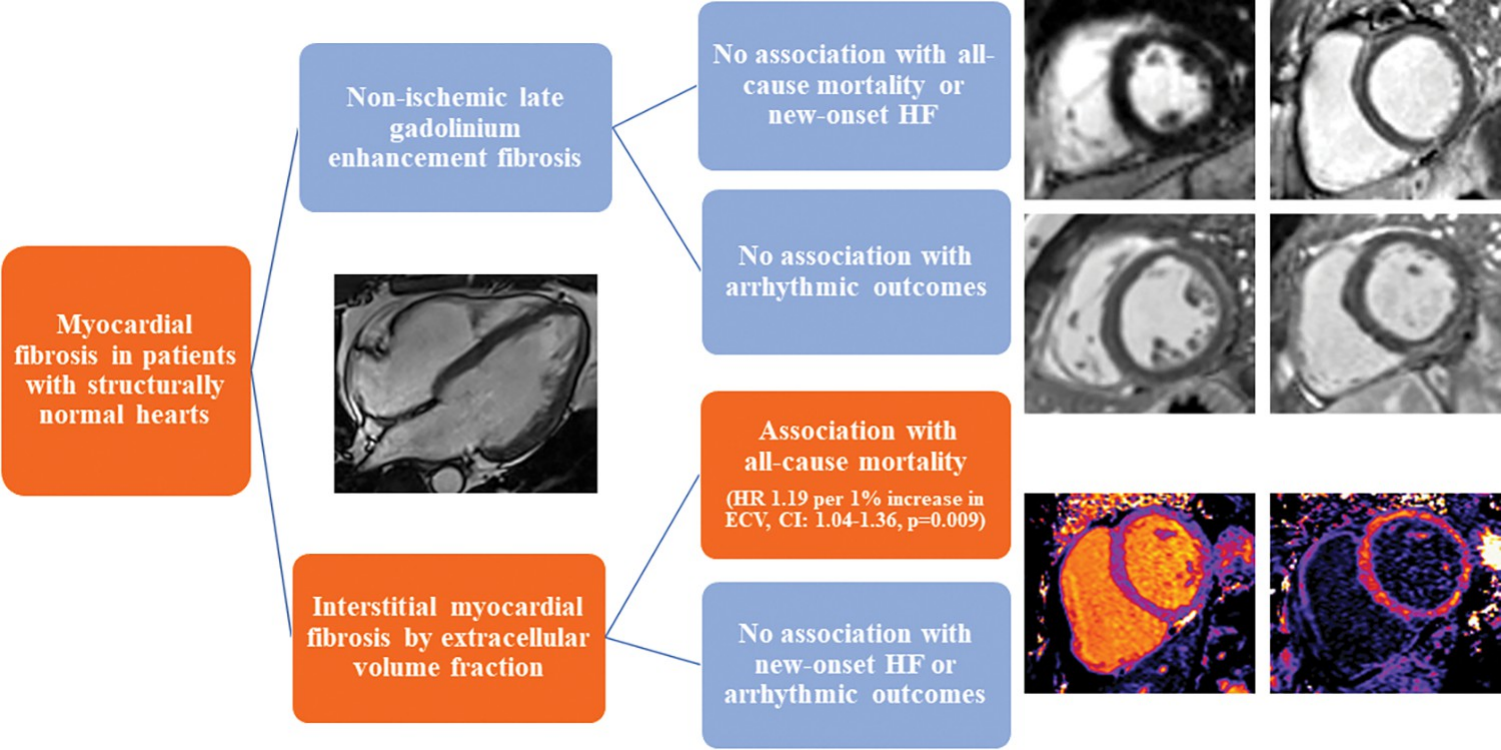
Myocardial fibrosis on cardiovascular magnetic resonance and its association with prognosis in patients with structurally normal hearts. Abbreviations: HF–heart failure, HR–hazard ratio, ECV—extracellular volume fraction. CI–confidence interval.

We investigated the prognostic significance of myocardial fibrosis in patients with structurally normal hearts. Our data suggests that nonischemic LGE (including isolated RVIP fibrosis) is a relatively common finding in patients referred for CMR with structurally normal hearts. We note that LGE presence, pattern, location, and extent were not associated with all-cause mortality, new-onset HF or arrhythmic events, over a 5-year follow-up. ECV, on the other hand, was a predictor of all-cause mortality in patients with structurally normal hearts (Fig 4).

Our findings regarding nonischemic LGE are in agreement with recently published data in similar lower risk cohorts. In a study by Lota and colleagues with similar exclusion criteria and similar prevalence of nonischemic LGE (53.6%), mortality was not driven by the presence or extent of nonischemic LGE in patients with normal LV volumes and LVEF [3]. The study cohort was more inclusive, but the authors did not include RVIP as a form of nonischemic fibrosis, and did not report T1 mapping or ECV data [3]. RVIP, previously thought to be benign, has been shown to be an independent predictor of a combined outcome of HF admission or all-cause mortality in patients with dilated cardiomyopathy [10, 26, 27]. In another study midwall, subepicardial, and patchy LGE was associated with adverse outcomes (time to death or HF hospitalization), whereas such correlation was not demonstrated for RVIP fibrosis [4]. The population of that study, however, involved older adults with inclusion of patients with LV hypertrophy, depressed LVEF, and history of myocardial infarction [4]. Of note, the lack of association of LGE with outcomes in our study remained when isolated RVIP fibrosis was excluded from the analysis.

Nonischemic LGE is thought to be multifactorial and may arise from a variety of conditions [2]. RVIP fibrosis may be a consequence of aging, right ventricular overload, or can be seen in athletes [26, 28]. We note that RVIP fibrosis was common in our relatively young cohort despite exclusion of patients with valvular heart disease and pulmonary hypertension. Septal and inferolateral LGE may suggest a preclinical stage of disease [29]. It has been suggested that septal LGE in patients with structurally normal hearts may represent a preclinical stage of dilated cardiomyopathy [30]. Inferolateral wall LGE may relate to diabetes, prior myocarditis, or a preclinical phase of cardiomyopathies [3, 31, 32]. Nonischemic LGE is also a common finding in patients with systemic hypertension and may be useful in identifying patients at risk of diastolic HF [33–35].

We demonstrate that ECV is a predictor of all-cause mortality in a relatively young cohort with structurally normal hearts. Although patients with structural heart abnormalities were excluded from the analysis, the association between all-cause mortality and ECV was present. Since ECV is a reliable estimate of interstitial fibrosis particularly in the absence of inflammation, strict definition of our study group makes this risk stratifier more specific [12]. ECV is believed to serve as the outcome from downstream impact of comorbidities and clinical risk and may represent an early “pre-clinical” marker of disease [17]. Interstitial expansion as measured by ECV has been shown to be present in multiple disease states spanning a broad range of severity from asymptomatic phenotype negative patients with a genetic predisposition to cardiac disease, to those with end stage cardiomyopathies [36]. ECV expansion may precede significant increase of LV mass in patients with hypertension and normal LV mass [28]. Interstitial MF has been shown to be associated with aging and diabetes [2, 11, 16]. In patients at risk of HF, ECV has been shown to be a validated risk stratification tool in predicting HF hospitalization and all-cause mortality [37]. In the current era of personalized medicine and earlier disease identification, ECV holds promise as a biomarker of risk. A comparison of ECV and global longitudinal strain (GLS), both thought to be earlier markers of disease, found that ECV and GLS correlate only minimally thus likely representing distinct domains of cardiac vulnerability, but both are independently associated with all-cause mortality, with ECV providing incremental prognostic value [38]. These data suggest that ECV may assess different metrics from clinical and standard imaging parameters and may represent an independent risk stratifier. Our cohort is representative of the national prevalence of risk factors associated with interstitial expansion such as diabetes and hypertension [39, 40], thus supporting its clinical applicability. We demonstrate an independent association of ECV and all-cause mortality and highlight the potential for the prognostic nature of interstitial expansion.

Our study supports recent data that the presence of nonischemic LGE in patients with structurally normal hearts may not require further testing or tailored therapy in absence of other risk factors [3]. A more aggressive approach should be implemented in cases of abnormal LV structure, increased wall thickness, depressed LVEF, or genetic/clinical predisposition [3]. Interstitial fibrosis by ECV, on the other hand, may represent an early stage of disease and hence a potential therapeutic target. It has been previously shown that renin-angiotensin-aldosterone system inhibition was associated with lower ECV in patients with higher disease severity [11, 41]. Further data now support the reversible nature of interstitial expansion as measured by ECV in both hypertensive heart disease [42] and HF with preserved EF [43], thus highlighting the importance of ECV as a potentially *actionable* risk stratification tool.

### Limitations

This is a single center study which should be validated in large multicenter cohorts. Given the strict criteria applied to define our study group, the findings are representative of a relatively younger lower risk population with preserved LVEF and no structural heart disease. LGE analysis did not involve assessment of LGE percentage of myocardial mass; however, the assessment of LGE extent via the number of involved AHA segments has been well established. T1 mapping was not routinely performed in all patients at our institution during the study dates, particularly prior to 2014 hence limiting our T1 mapping sample size to approximately half of the whole cohort. Additionally, during the study period only mid-short axis T1 maps were obtained routinely, thus limiting coverage. Given the retrospective nature of the study involving review of the electronic medical record, the precise cause of death could not be determined in all patients. Due to this limitation, all-cause mortality was used as the endpoint. Given the relatively small sample size and low event rate, our single center study may be underpowered to detect a small difference in the incidence of events. Therefore, our findings should be validated in larger, multicenter cohorts with longer follow-up.

## Conclusions

Our data demonstrates that nonischemic LGE is a common finding in patients with preserved LVEF and no structural heart abnormalities and is not associated with all-cause mortality, new-onset HF or arrhythmic outcomes. However, interstitial microscopic fibrosis by ECV is independently associated with all-cause mortality in patients with preserved LVEF and no structural heart abnormalities. Interstitial fibrosis may represent a pre-clinical phenotype and perhaps warrants stricter surveillance and possible early initiation of pharmacotherapy.

## Supporting information

S1 Table Utilized cardiovascular magnetic resonance sequences. Abbreviations: VLA, vertical long axis; HLA, horizontal long axis; 3CH, three chamber long axis, SAX, short axis; MOLLI, Modified Look-Locker Inversion Recovery; ROI, region of interest; LGE, late gadolinium enhancement; AHA, American Heart Association.
(DOCX)

S2 Table Association between number of LGE segments and clinical outcomes. Abbreviations: HF, Heart Failure; LGE, late gadolinium enhancement.
(DOCX)

S3 Table Association between LGE location and clinical outcomes. Abbreviations: HF, Heart Failure; LGE, late gadolinium enhancement. * Cannot be calculated as there are no New-onset HF or Arrhythmic Events in the Inferolateral Wall Group.
(DOCX)

S4 Table Association of ECV and all-cause mortality in different multivariable models. Abbreviations: HR, hazard ratio; CI, confidence interval; ECV, extracellular volume; LVEF, left ventricular ejection fraction; LGE, late gadolinium enhancement.
(DOCX)

S1 Fig The optimal ECV cutoff for worse overall survival outcomes using maximally selected rank statistics. (DOCX)

S2 Fig Testing for the proportional hazard assumption for multivariable model. The proportional hazard assumption is supported by statistical tests and graphical diagnostics using scaled Schoenfeld residuals.
(DOCX)
