# Supplementary material for: Risk stratification in patients with structurally normal hearts: Does fibrosis type matter?
Source: PLoS One. 2023 Dec 20;18(12):e0295519. doi: 10.1371/journal.pone.0295519 (PMC10732365; doi:10.1371/journal.pone.0295519)
Supplement: S1 Table — Abbreviations: VLA, vertical long axis; HLA, horizontal long axis; 3CH, three chamber long axis, SAX, short axis; MOLLI, Modified Look-Locker Inversion Recovery; ROI, region of interest; LGE, late gadolinium enhancement; AHA, American Heart Association. (DOCX) [file pone.0295519.s001.docx]

**Risk stratification in patients with structurally normal hearts: Does fibrosis type matter?**

**Corresponding author: Karolina M. Zareba**

**Supporting Information**

**Supplemental Table 1. Utilized cardiovascular magnetic resonance sequences.**

| **Sequence** | **Imaging planes** | **Analysis** |
| --- | --- | --- |
| Cine: balanced steady-state free precession | VLA, HLA, 3CH, SAX stack | Standard volumetric analysis |
| Pre-contrast T1 map: MOLLI 5(3)3 | mid SAX | Septal ROI with care to avoid partial volume effects |
| Post-contrast T1 map: MOLLI: 4(1)3(1)2 | mid SAX | Septal ROI with care to avoid partial volume effects |
| LGE: phase sensitive inversion recovery | VLA, HLA, 3CH, SAX stack | LGE presence, assessed by two expert level 3 operators blinded to clinical data, present in either two contiguous slices or two orthogonal planes. LGE extent evaluated utilizing the AHA segment model. |

**Abbreviations:** VLA, vertical long axis; HLA, horizontal long axis; 3CH, three chamber long axis, SAX, short axis; MOLLI, Modified Look-Locker Inversion Recovery; ROI, region of interest; LGE, late gadolinium enhancement; AHA, American Heart Association.
