# Supplementary material for: Risk stratification in patients with structurally normal hearts: Does fibrosis type matter?
Source: PLoS One. 2023 Dec 20;18(12):e0295519. doi: 10.1371/journal.pone.0295519 (PMC10732365; doi:10.1371/journal.pone.0295519)
Supplement: S2 Table — Abbreviations: HF, Heart Failure; LGE, late gadolinium enhancement. (DOCX) [file pone.0295519.s002.docx]

**Risk stratification in patients with structurally normal hearts: Does fibrosis type matter?**

**Corresponding author: Karolina M. Zareba**

**Supporting Information**

**Supplemental Table 2. Association between number of LGE segments and clinical outcomes.**

|  | **HR** | **95% CI** | **P value** |
| --- | --- | --- | --- |
| ***Total number of LGE segments within the LGE positive group*** | | | |
| All-cause Mortality | 1.22 | 0.87-1.71 | 0.26 |
| New-onset HF | 0.85 | 0.52-1.40 | 0.52 |
| Arrhythmic Outcome | 0.93 | 0.67-1.29 | 0.65 |

Abbreviations: HF, Heart Failure; LGE, late gadolinium enhancement.
