# Supplementary material for: Risk stratification in patients with structurally normal hearts: Does fibrosis type matter?
Source: PLoS One. 2023 Dec 20;18(12):e0295519. doi: 10.1371/journal.pone.0295519 (PMC10732365; doi:10.1371/journal.pone.0295519)
Supplement: S3 Table — Abbreviations: HF, Heart Failure; LGE, late gadolinium enhancement. * Cannot be calculated as there are no New-onset HF or Arrhythmic Events in the Inferolateral Wall Group. (DOCX) [file pone.0295519.s003.docx]

**Risk stratification in patients with structurally normal hearts: Does fibrosis type matter?**

**Corresponding author: Karolina M. Zareba**

**Supporting Information**

**Supplemental Table 3. Association between LGE location and clinical outcomes.**

|  | **All-Cause Mortality** | **P value** | **New-onset HF** | **P value** | **Arrhythmic Outcome** | **P value** |
| --- | --- | --- | --- | --- | --- | --- |
| Septum | Ref |  | Ref |  |  |  |
| Inferolateral Wall | 1.83 (0.21-15.70) | 0.58 | -* |  | -* |  |
| Septum + Inferolateral Wall | 1.02 (0.20-5.26) | 0.98 | 1.30 (0.24-7.13) | 0.76 | 1.16 (0.36-3.78) | 0.80 |

Abbreviations: HF, Heart Failure; LGE, late gadolinium enhancement.

^*^ Cannot be calculated as there are no New-onset HF or Arrhythmic Events in the Inferolateral Wall Group.
