# Supplementary material for: Risk stratification in patients with structurally normal hearts: Does fibrosis type matter?
Source: PLoS One. 2023 Dec 20;18(12):e0295519. doi: 10.1371/journal.pone.0295519 (PMC10732365; doi:10.1371/journal.pone.0295519)
Supplement: S4 Table — Abbreviations: HR, hazard ratio; CI, confidence interval; ECV, extracellular volume; LVEF, left ventricular ejection fraction; LGE, late gadolinium enhancement. (DOCX) [file pone.0295519.s004.docx]

**Risk stratification in patients with structurally normal hearts: Does fibrosis type matter?**

**Corresponding author: Karolina M. Zareba**

**Supporting Information**

**Supplemental Table 4.** **Association of ECV and all-cause mortality in different multivariable models.**

|  | **Model 1** | | | **Model 2** | | | **Model 3** | | |
| --- | --- | --- | --- | --- | --- | --- | --- | --- | --- |
|  | **HR** | **95% CI** | **P value** | **HR** | **95% CI** | **P value** | **HR** | **95% CI** | **P value** |
| ECV (per 1%) | 1.22 | 1.04-1.44 | 0.015 | 1.21 | 1.03-1.44 | 0.02 | 1.24 | 1.04-1.49 | 0.017 |
| Age (per 1 year) | 1.03 | 0.96-1.10 | 0.42 | 1.02 | 0.96-1.09 | 0.55 | 1.04 | 0.97-1.11 | 0.33 |
| Male | 0.89 | 0.10-7.72 | 0.91 | 0.99 | 0.11-8.74 | 0.99 | 0.67 | 0.08-5.92 | 0.72 |
| Hypertension | 3.94 | 0.71-21.95 | 0.12 | 3.53 | 0.64-19.56 | 0.15 | 2.75 | 0.47-15.95 | 0.26 |
| LVEF (per 1%) | - | - | - | 1.09 | 0.99-1.20 | 0.08 | - | - | - |
| LGE presence | - | - | - |  |  |  | 3.98 | 0.41-38.98 | 0.24 |

Abbreviations: HR, hazard ratio; CI, confidence interval; ECV, extracellular volume; LVEF, left ventricular ejection fraction; LGE, late gadolinium enhancement.
