# Supplementary material for: Risk stratification in patients with structurally normal hearts: Does fibrosis type matter?
Source: PLoS One. 2023 Dec 20;18(12):e0295519. doi: 10.1371/journal.pone.0295519 (PMC10732365; doi:10.1371/journal.pone.0295519)

**Risk stratification in patients with structurally normal hearts: Does fibrosis type matter?**

**Corresponding author: Karolina M. Zareba**

**Supporting Information**

**Supplemental Figure 1. The optimal ECV cutoff for worse overall survival outcomes using maximally selected rank statistics.**


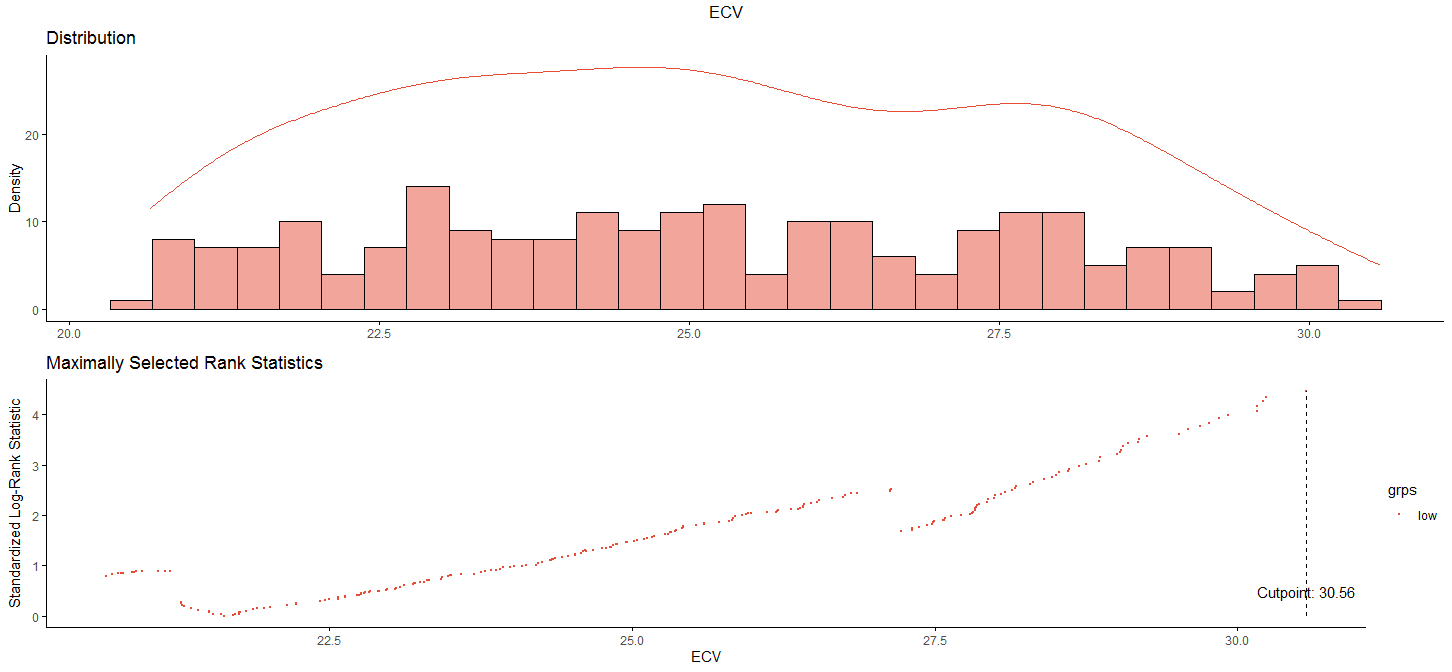

Supplement: S1 Fig — (DOCX) [file pone.0295519.s005.docx]
