# Supplementary material for: Risk stratification in patients with structurally normal hearts: Does fibrosis type matter?
Source: PLoS One. 2023 Dec 20;18(12):e0295519. doi: 10.1371/journal.pone.0295519 (PMC10732365; doi:10.1371/journal.pone.0295519)
Supplement: S2 Fig — The proportional hazard assumption is supported by statistical tests and graphical diagnostics using scaled Schoenfeld residuals. (DOCX) [file pone.0295519.s006.docx]

**Risk stratification in patients with structurally normal hearts: Does fibrosis type matter?**

**Corresponding author: Karolina M. Zareba**

**Supporting Information**

**Supplemental Figure 2.** **Testing for the proportional hazard assumption for multivariable model.**


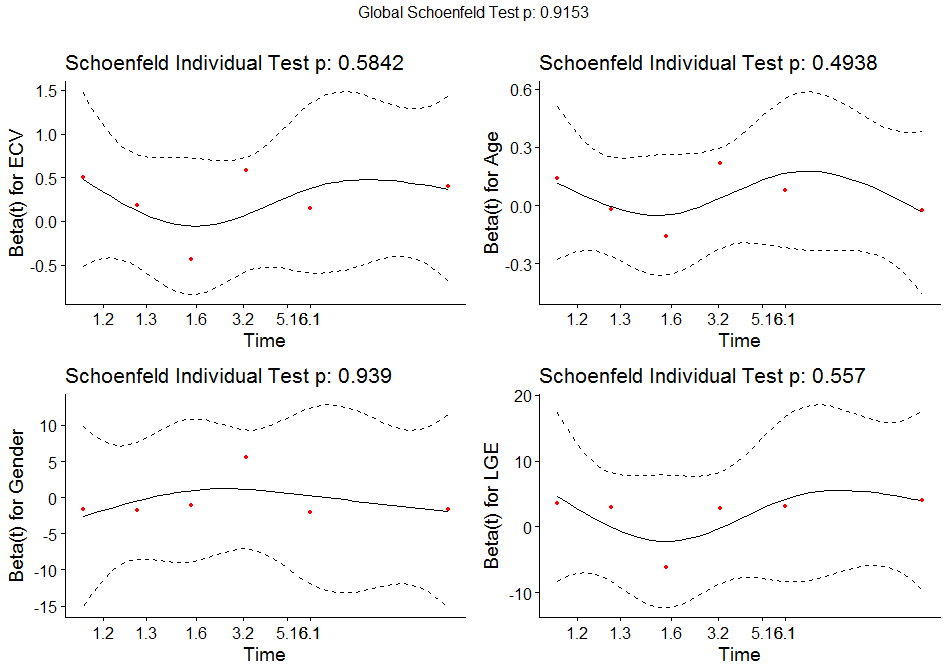


The proportional hazard assumption is supported by statistical tests and graphical diagnostics using scaled Schoenfeld residuals.
